# Genome-Wide Association Studies Reveal a Simple Genetic Basis of Resistance to Naturally Coevolving Viruses in *Drosophila melanogaster*

**DOI:** 10.1371/journal.pgen.1003057

**Published:** 2012-11-15

**Authors:** Michael M. Magwire, Daniel K. Fabian, Hannah Schweyen, Chuan Cao, Ben Longdon, Florian Bayer, Francis M. Jiggins

**Affiliations:** Department of Genetics, University of Cambridge, Cambridge, United Kingdom; University of Florida, United States of America

## Abstract

Variation in susceptibility to infectious disease often has a substantial genetic component in animal and plant populations. We have used genome-wide association studies (GWAS) in *Drosophila melanogaster* to identify the genetic basis of variation in susceptibility to viral infection. We found that there is substantially more genetic variation in susceptibility to two viruses that naturally infect *D. melanogaster* (DCV and DMelSV) than to two viruses isolated from other insects (FHV and DAffSV). Furthermore, this increased variation is caused by a small number of common polymorphisms that have a major effect on resistance and can individually explain up to 47% of the heritability in disease susceptibility. For two of these polymorphisms, it has previously been shown that they have been driven to a high frequency by natural selection. An advantage of GWAS in *Drosophila* is that the results can be confirmed experimentally. We verified that a gene called *pastrel*—which was previously not known to have an antiviral function—is associated with DCV-resistance by knocking down its expression by RNAi. Our data suggest that selection for resistance to infectious disease can increase genetic variation by increasing the frequency of major-effect alleles, and this has resulted in a simple genetic basis to variation in virus resistance.

## Author Summary

In most animal populations, individuals vary genetically in how susceptible they are to infectious disease. To understand the genetic basis of this variation, we have infected a panel of inbred lines of the fruit fly *D. melanogaster* with viruses and have looked for genetic variants associated with resistance to infection. Using two viruses that naturally infect this species, we found a high level of genetic variation, much of which is due to a small number of genetic variants that have a large effect on virus resistance. Previous work has shown that two of these variants resulted from recent mutations that increased resistance and have been driven to a high frequency by natural selection. Furthermore, we did not find similar major-effect variants when we infected flies with viruses isolated from other species of insects. Therefore, selection for virus resistance appears to increase genetic variation in susceptibility to viral infection. Understanding the function of the genes, we have identified promises to give new insights into the antiviral defences of insects.

## Introduction

Variation in susceptibility to infectious disease often has a substantial genetic component in animal and plant populations [1]–[4]. As pathogens are a powerful selective force in the wild, natural selection is expected to play an important role in determining the nature of this genetic variation. Selection for resistance to infectious disease can change rapidly, as new pathogens appear in the population [5], or existing pathogens evolve, for example to evade or sabotage host defences [6]. This selection can result both in positive selection increasing the frequency of mutations that generate new resistance alleles [7], [8], and balancing selection stably maintaining resistant and susceptible alleles of a gene [9].

Over the last decade, genome-wide association studies (GWAS) have provided a more complete picture of the genetic architecture of disease susceptibility [10]. The majority of these studies have investigated non-communicable diseases in humans, and while many polymorphisms associated with susceptibility have been identified, these often have small effects and together can explain only a small proportion of the heritability [11] (but see [12]). It has been suggested that the reason for this is that new mutations that increase susceptibility to non-communicable disease may tend to be deleterious, so alleles that have large effects are either removed from the population or kept at a low frequency by purifying selection [11]. However, both GWAS and classical linkage mapping studies suggest that the genetic architecture of infectious disease susceptibility may be qualitatively different [13], as major effect polymorphisms that protect hosts against infection have been identified in many organisms, including plants [11], humans [3], [13] and insects [7], [14]. Furthermore, these polymorphisms are often under strong positive or balancing selection [7]–[9]. It has therefore been argued that natural selection may cause the variation in infectious disease susceptibility to have a simpler genetic architecture than non-communicable diseases as major-effect alleles can reach a higher frequency in populations [13].

In arthropods, several studies suggest that susceptibility to infectious disease may often be affected by major-effect polymorphisms (e.g.[7], [14], [15]). In *Drosophila melanogaster*, linkage mapping has been used to identify major effect resistance polymorphisms that affect susceptibility to both the sigma virus (DMelSV) [16], [17] and parasitoid wasps [18]. In the case of DMelSV, two of these loci have been identified at the molecular level (*ref(2)P* and *CHKov1*), and they have been found to be common in natural populations [7], [14], [19]. In addition, polymorphisms in known immunity genes have been found to affect susceptibility to bacterial infection, and some of these have substantial effects [2], [20], [21].

To understand how natural selection affects the genetics of disease susceptibility, we have used GWAS to examine the effects of selection for resistance to pathogens on patterns of genetic variation. To do this we infected *D. melanogaster* both with viruses that naturally occur in this species and viruses isolated from other species. The two of the viruses that naturally infect *D. melanogaster* are Drosophila C Virus (DCV), which is a positive sense RNA virus in the Dicistroviridae that infects a range of *Drosophila* species [22], [23], and the sigma virus DMelSV, which is a rhabdovirus that is a specialist on *D. melanogaster* [16]. The other two viruses naturally infect other insect species are DAffSV, which is another sigma virus that naturally infects *Drosophila affinis* [24], [25] and Flock House Virus (FHV), which is a nodavirus that was isolated from beetles but can infect an extremely broad range of organisms [26]. We found that the heritability of susceptibility to the two natural *D. melanogaster* viruses is high due to a small number of common major-effect polymorphisms. In contrast there is less genetic variation in susceptibility to viruses isolated from other species, and here there is no evidence of major effect polymorphisms.

## Results

### Genetic variation in virus resistance

To investigate genetic variation in resistance to viruses, we injected 47,220 flies from 185 different inbred lines from the Drosophila Genetic Reference Panel (DGRP) with four different viruses (Table 1; note that the DMelSV data, but not this analysis, has been published before [7]). The extent of genetic variation in susceptibility varied considerably between the different viruses, with the greatest genetic variation being present when flies are exposed to viruses that infect *D. melanogaster* in nature. Comparing the two viruses where resistance was measured in terms of survival time—DCV and FHV—we found DCV resistance has significantly greater heritability (Table 1). When the two sigma viruses, DMelSV and DAffSV, are compared, again the heritability is significantly greater in resistance to the naturally occurring virus DMelSV (Table 1). While differences in heritability can be caused by differences in genetic or environmental variation, it is clear that there is genetic variation in resistance to the natural pathogens of *D. melanogaster*. In the case of DCV and FHV, DCV has the greater coefficient of genetic variation (Table 1; *CV_g_*) [27]. It is not possible to calculate the coefficient on variation for the sigma virus data as it is analysed on a logit scale. However, by inspecting *V_e_* and *V_g_* in Table 1, it is clear that the differences in the heritability of resistance to DMelSV and DAffSV are primarily driven by differences in *V_g_*.

**Table 1:** Genetic variation in susceptibility to four different viruses.

Virus	Natural host	Trait	*N* flies	*N* lines	*V_e_*	*V_g_*	*h^2^*	*CV_g_*
DCV	*D. melanogaster*	Survival	14,415	185	1.15 (1.13–1.19)	0.61 (0.49–0.74)	0.34 (0.30–0.39)	20 (18–22)
FHV	Beetle	Survival	12,660	182	2.10 (2.03–2.18)	0.17 (0.13–0.23)	0.07 (0.05–0.10)	7 (6–8)
DMelSV	*D. melanogaster*	CO_2_ sensitivity	11,541	185	4.79 (4.50–5.08)	1.94 (1.47–2.41)	0.29 (0.24–0.34)	-
DAffSV	*D. affinis*	CO_2_ sensitivity	8,604	181	3.88 (3.69–4.03)	0.61 (0.43–0.78)	0.13 (0.10–0.16)	-

Genetic variation is expressed as heritability (*h^2^*) and the coefficient of genetic variation (*CV_g_*), and 95% credible intervals are given in parentheses. The natural host is the insect from which the virus was isolated. Flies were classed as infected with DMelSV and DAffSV if they were paralysed after exposure to CO_2_. *CV_g_* was not calculated when the data was ratios of dead and alive flies analysed on a logit scale. *V_g_* is genetic variance and *V_e_* is the environmental variance.

In all cases the genetic correlation in the level of resistance to different viruses is low, indicating that different genes are controlling resistance to different viruses (Table 2). In particular, the sigma viruses (DMelSV and DAffSV) showed no evidence of any genetic correlation, despite being relatively closely related [24], [25]. Despite being small, there is a significant positive genetic correlation in susceptibility between three pairs of viruses, indicating that there may be some variation in the ability to survive viral infection in general. The low genetic correlations also confirm that we are measuring susceptibility to the different viruses and not an artefact of the injection procedure.

**Table 2:** Genetic correlations in susceptibility to four different viruses.

Viruses	Genetic Correlation
DCV- FHV	0.23 (0.05, 0.40)*
DCV- DMelSV	0.27 (0.12, 0.42)*
DCV- DAffSV	−0.05 (−0.22, 0.14)
FHV- DMelSV	0.15 (−0.02, 0.35)
FHV- DAffSV	0.22 (0.02, 0.43)*
DMelSV- DAffSV	−0.09 (−0.28, 0.08)

95% credible intervals are given in parentheses.

* Represent MCMC *P-*values<0.05.

### Resistance to viruses that infect *D. melanogaster* in the wild has a simple genetic basis

**Figure 1:**
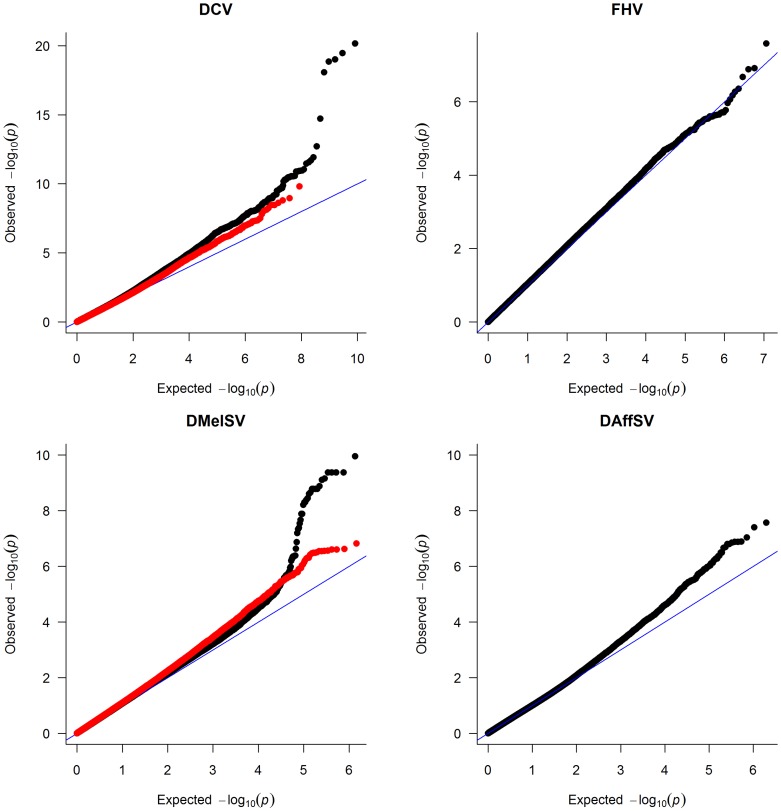
Quantile–quantile plots of *P-*values. The black dots represent the observed *P-*values against the *P-*values that are expected under the null hypothesis (that there are no true associations with resistance to four viruses), and the straight line is the distribution expected if the observed values equal the expected values. The red points show the *P-*values after the effect of the polymorphisms in *pastrel* (DCV), *ref(2)P* (DMelSV) and *CHKov1* (DMelSV) have been accounted for. The null distribution of expected *P*-values was obtained by permutation.

To identify polymorphisms that are associated with resistance to the four viruses, we performed genome-wide association studies using the published genome sequences of the DGRP lines [28]. To correct for multiple tests and obtain a genome-wide significance threshold, we permuted the trait data across the lines and repeated the GWAS 400 times, each time recording the lowest *P-*value across the entire genome. Quantile-quantile (qq) plots of the *P-*values show that there are highly significant associations in the experiments using DCV and DMelSV — the two viruses that infect *D. melanogaster* in the wild — but not in the experiments using FHV and DAffSV (Figure 1).

**Figure 2:**
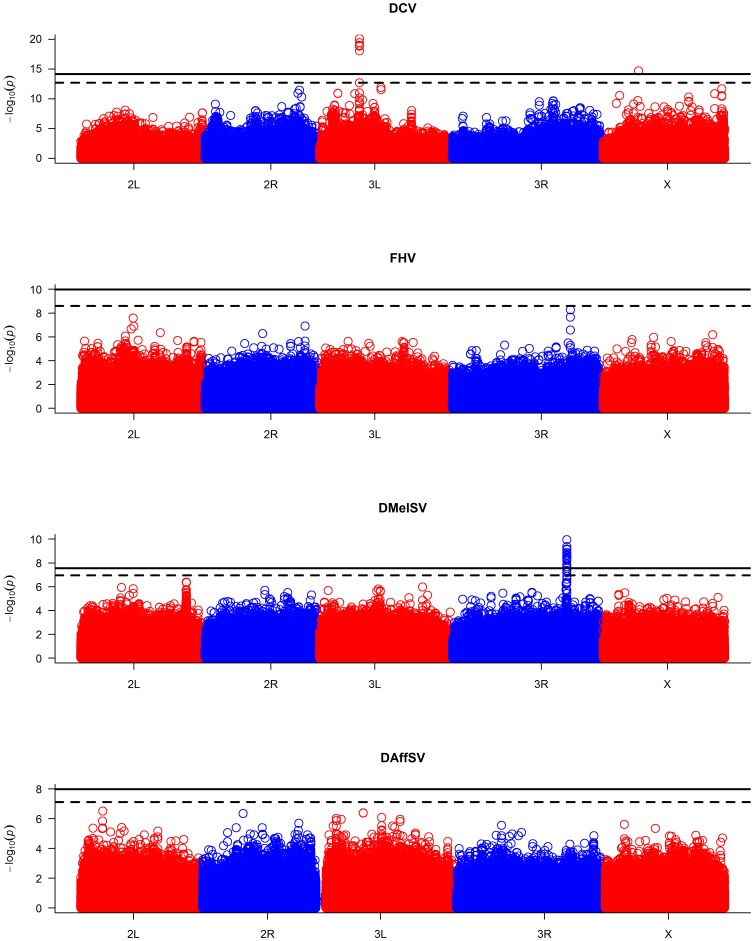
Manhattan plots of the *P*-values for the association between SNPs and virus resistance. The horizontal lines are genome-wide significance thresholds of *P* = 0.05 (solid line) and *P* = 0.2 (dashed line) that were obtained by permutation. The five chromosome arms are different colours.

When the *P-*values are plotted along the chromosomes, it is clear that the most significant *P-*values cluster together (Figure 2, Figure S1). In the case of DMelSV there is a cluster of significant SNPs around *CHKov1* on chromosome arm 3R, which is a gene where we have previously shown that a transposable element insertion is associated with resistance to this virus [7] (Figure 2, Figure S1). The second most significant cluster of SNPs in this experiment falls just below the genome-wide significance threshold, and is on chromosome arm 2L (Figure 2, Figure S1). The SNPs in this cluster are all in strong linkage disequilibrium with a polymorphism in *ref(2)P* that is known to cause resistance (the causal polymorphism was genotyped by PCR and included in this analysis),[8], [14]. In the case of DCV there is a cluster of significant SNPs in and around a gene on chromosome arm 3L called *pastrel*, which has not previously been implicated in antiviral defence. There were no significant associations with susceptibility to FHV or DAffSV using a genome-wide significance threshold of *P*<0.05 (Figure 2, Figure S1).

We repeated the GWAS accounting for the effects of the polymorphisms in *ref(2)P*, *CHKov1* and *pastrel*. The quantile-quantile plots of the resulting *P-*values (Figure 1, red points) show that these genes can account for all of the large excess of highly significant associations with DCV and DMelSV resistance. The resulting distribution of *P*-values resembles that seen for the other two viruses that do not naturally infect *D. melanogaster*.

To investigate how much of the genetic variation in susceptibility is explained by our GWAS, we calculated the proportion of the heritability that is explained by these genes. Assuming the polymorphisms have additive effects, then their contribution to additive genetic variation is 2*pqa* ^2^, where *p* and *q* are the frequencies of the alleles, and *a* and −*a* are the genotypic values of the resistant and susceptible homozygotes. In the case of DCV, *pastrel* (3L:7350895) can explain 47% of the heritability. In the case of DMelSV, *ref(2)P* explains 8% of the heritability, the *doc* element insertion in *CHKov1* explains 29% of the heritability, and in combination these polymorphisms explain 37% of the heritability.

The proportion of the heritability explained by these polymorphisms may be biased by two factors. First, we are injecting the virus, which is an unnatural route of infection, and in the case of the sigma viruses we are assaying a symptom of infection rather than viral titres or effects on host survival. Second, we can only estimate the amount of genetic variation explained in inbred lines, and this can only be directly extrapolated to outcrossed populations if all the genes affecting resistance are additive. Unfortunately, we cannot estimate the importance or direction of this bias as we only used inbred lines (the only one of the three genes for which levels of dominance has been investigated is *ref(2)P*, where heterozygotes have intermediate levels of resistance when injected with the virus [29]). However, the bias could be substantial if we make the extreme assumptions about dominance. If the susceptible *pastrel* allele is recessive and only half the remaining genetic variance is additive, this polymorphism will explain 84% of *V_a_* in an outcrossed population. Conversely, if the resistant *pastrel* allele is fully dominant and all the remaining genetic variance is additive, this polymorphism will explain just 7% of *V_a_* in an outcrossed population.

In addition to these major-effect polymorphisms, there were also other suggestive results. The most significant association for DAffSV was a synonymous SNP in *scavenger receptor C1* (2L:4123156 A/T; individual *P* = 6.41×10^−8^; genome-wide permutation *P* = 0.18). This gene functions both as a pattern recognition receptor of bacteria [30] and allows the uptake of dsRNA into cells [31]. A polymorphism in the gene *Anaphase promoting complex 7* was associated with a 3.7 day increase in survival after injection with DCV (X:6491634 G/T; individual *P* = 1.95×10^−15^; genome-wide permutation *P*<0.05). This was at a low frequency, with the resistant variant present in 4 of 145 lines. Furthermore, it is a synonymous polymorphism, suggesting that it may not be a causal variant. The QQ plots also show that there is an excess of small *P-*values in three of the analyses (Figure 1), suggesting that there may be many more polymorphisms to be discovered, or that there is some unidentified population stratification.

As the polymorphisms in *pastrel*, *CHKov1* and *ref(2)P* have a large effect on resistance, we repeated the GWAS taking account of these polymorphisms by including them as fixed effects in the model. However, this did not lead to the identification of additional SNPs associated with resistance (Figure S2). The most significant association with DCV resistance remained *Anaphase promoting complex 7* (X:6491634 G/T). For DMelSV it was a SNP in the intron of *off-track* (2R:7899322 A/T, genome-wide *P* = 0.29), which is a transmembrane receptor that controls a variety of developmental and physiological processes [32].

### Resistance genes are specific to different viruses

**Figure 3:**
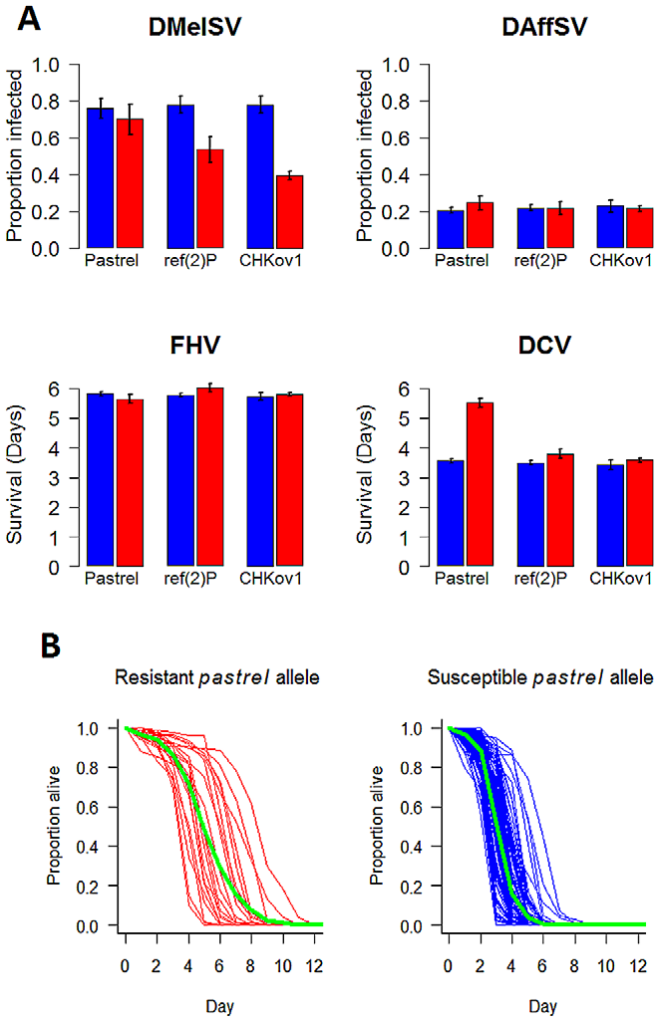
The effect of the three polymorphisms affecting susceptibility on four different viruses. In Panel A, blue bars are the susceptible allele and red bars the resistant allele. The estimates and standard errors were obtained using a general linear mixed model with the mean survival or proportion infected in each vial as the response. Significant associations are controlled for when estimating the effects of other genes, and the estimates assume that the flies have the susceptible allele of other genes. Panel B, shows the survival curves of lines with the two alleles of *pastrel* (3L:7350895 Ala/Thr). The green lines show the combined survival of all the flies with each allele (combined across the different lines).

The polymorphisms in *pastrel*, *CHKov1* and *ref(2)P* have highly specific effects, altering susceptibility to just one of the four viruses (Figure 3). Against these target viruses, the effect on the susceptibility of individual flies is considerable (Figure 3). Comparing flies that are homozygous for the resistant and susceptible alleles, the most significant SNP in *pastrel* increases survival times by 55%. The *doc* element insertion in *CHKov1* reduces the proportion of infected flies by 39%, while the *ref(2)P* polymorphism is associated with a 24% reduction in infection rates (see also [7]). When large numbers of statistical tests are performed and the statistical power is low, as is the case in many genetic association studies, there is a tendency to overestimate effect sizes [33]. However, the extremely low *P-*values associated with our resistance genes suggest our statistical power was high and therefore these effect size estimates are reliable.

### *pastrel* confers resistance to DCV

In *pastrel* there are six SNPs that are associated with resistance to DCV at *P*<10^−12^. These include two adjacent SNPs in the 3′UTR (genome positions: 3L:7350452 T/G, 3L:7350453 A/G), two non-synonymous SNPs (3L:7350895 Ala/Thr, 3L:7352880 Glu/Gly) and two SNPs in introns (3L:7351494 C/T, 3L:7352966 T/G). All of these are in linkage disequilibrium, with the two SNPs in the 3′ UTR being perfectly associated (these are therefore considered as a single variant in subsequent analyses). To try and disentangle which of the polymorphisms might be a causal variant, we fitted a general linear mixed-effects model in which all five variants were included as fixed effects. This allows us to calculate the marginal significance of each polymorphism (i.e. the *P-*value after controlling for the effects of all the other SNPs). In this analysis only a non-synonymous SNP in the last coding exon of the *pastrel* remained highly significant (3L:7350895: *F*_1,116_ = 18.2, *P*<0.0001; all other *P-*values>0.01). This SNP occurred in 21 of 142 lines that were sequenced at this site. We then tested the significance of each of the other four variants individually while controlling for the effects of 3L:7350895, by fitting general linear mixed-effects models and calculating sequential *P-*values from an ANOVA table. When we did this, all the other SNPs are significant (*P*<0.0001 in all cases). If we assume that we have included all the polymorphisms in this region in our analysis, this suggests that the non-synonymous polymorphism 3L:7350895 and at least one of the other sites are causal variants — but strong linkage disequilibrium prevents us from identifying which one(s). However, many polymorphisms, including indels, are missing from this dataset, so another polymorphism in this region that is not included in our analysis may be causing flies to be resistant to DCV.

**Figure 4:**
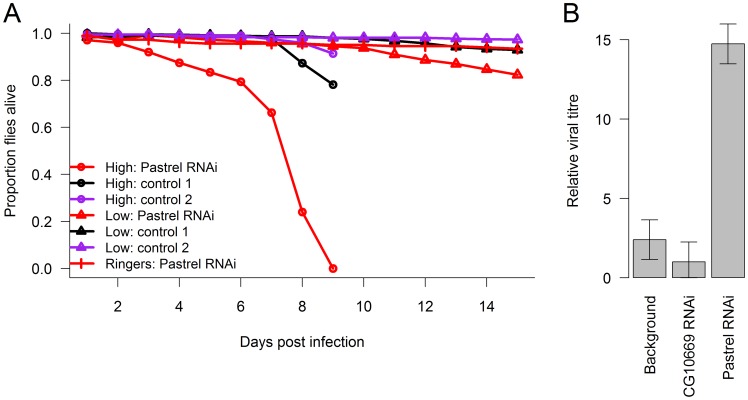
The effect of knocking down *pastrel* expression. The effect of knocking down *pastrel* expression on (A) the survival of infected flies with a high or low dose of DCV (see methods) and (B) viral titres 14 days post infection with the low dose. Control 1 were flies in which a gene unrelated to viral infection was knocked down (*CG10669*), and Control 2 were flies with the same genetic background as the *pastrel*-RNAi flies. Error bars are standard errors. Observations on the high dose treatment stopped on day 9.

To confirm the antiviral role of *pastrel*, we used RNAi to knock down the gene in flies that were homozygous for the susceptible allele. To do this, we expressed hairpin RNAs that target the *pastrel* gene under the control of a constitutively and ubiquitously expressed Gal4 driver. When the flies were infected with a high dose of DCV, this resulted in a large reduction in survival rates relative to both a control with a similar genetic background (Figure 4A; Cox proportional hazard mixed model: *z* = 3.62, *P* = 0.0003) and a control where we knocked down a gene unrelated to viral resistance (Figure 4A; Cox proportional hazard mixed model: *z* = 3.19 *P* = 0.001). To allow us to investigate viral titres, we also infected flies with a lower dose of DCV, which caused less mortality (Figure 4A). The viral titre in the flies where *pastrel* had been knocked down was ∼6 times greater than the background control and ∼15 times greater than the control gene (Figure 4B; *F_2,18_* = 23.3, *P* = 10^−5^).

## Discussion

We have found that a small number of major-effect polymorphisms can explain a substantial proportion of the genetic variation in the susceptibility of *D. melanogaster* to viral infection. These genes have either been previously identified by linkage mapping, or, in the case of *pastrel*, were verified by RNAi in this study. These polymorphisms are only seen when flies were infected with the two viruses that occur naturally in *D. melanogaster* populations — we were unable to detect any significant associations when using viruses that naturally infect other insects. The consequence of this is that the genetic variation in susceptibility to the naturally occurring viruses is substantially greater than to viruses from other species. Combined with previous data showing that two of these resistance alleles have been driven to a high frequency by positive selection [7], [8], [34], these results suggest that selection by viruses in natural populations may be increasing genetic variation in disease susceptibility. As the resistance alleles that we detected have highly specific effects against a single virus, genetic variation in susceptibility to infection by viruses isolated from other species of insects has remained low.

Our results support the suggestion that the genetic architecture of infectious disease susceptibility may be different from non-communicable diseases due to selection by parasites [13], [35]. GWAS in humans have mostly focused on non-communicable disease, and have tended to find polymorphisms of modest effect. In contrast, work on infectious disease in humans has described numerous loci with a major-effect on susceptibility [3], [13], [35], and similar patterns have been reported by QTL studies in other animals [36]. This has led to the suggestion that variation in pathogen resistance may often be controlled by a mixture of major-effect polymorphisms and other loci that are difficult to detect because they are rare or have small effects [13]. Our results corroborate this pattern, as while we find a few major-effect genes, over half of the total genetic variation remains unexplained. Furthermore, our results provide support for the role of natural selection by parasites in increasing the frequency and effect size of disease susceptibility loci. If this pattern proves to apply to other species, then GWAS on susceptibility to infectious disease promises to be a productive direction for future research.

Parasites can result both in balancing selection maintaining polymorphisms in host resistance, and directional selection, which will ultimately fix the resistant allele [6]. Previous work has shown that the resistant alleles of *CHKov1* and *ref(2)P* both arose recently by mutation and natural selection has caused them to increase in frequency [7], [8], [34], [37]. Therefore, it appears as though directional selection is driving new resistance genes through the population, and this is increasing genetic variation in disease susceptibility.

Directional selection on a trait can result in higher genetic variance when selection is acting on alleles that are initially at a low frequency [38]–[40]. If this is the case, selection will increase the frequency of rare alleles that previously contributed little to genetic variation in the population, and will therefore increase genetic variation in the trait [38]–[40]. For example, this process is thought to explain why selection by mate choice increases genetic variation in the cuticular hydrocarbons produced by *Drosophila serrata* [41]. In the case of the polymorphisms in *ref(2)P* and *CHKov1*, previous work has suggested that they have undergone a ‘hard’ selective sweep, where selection has been acting on new or rare polymorphisms [7], [8], [34], [37]. Therefore, they will have contributed little to genetic variation before selection, but now explain much of the heritability in this population. Certain traits, such as insecticide resistance, normally evolve in this way with selection acting on rare alleles [42]. This is thought to be because there are relatively few genetic changes that can cause insecticide resistance, and therefore there are too few mutations to generate much standing genetic variation [42]. If resistance to viruses also normally evolves due to selection on rare alleles, it may be common for directional selection to increase genetic variation. Fluctuating selection by parasites through time and space, or negatively frequency-dependent selection may also play an important role in increasing genetic variation.

In addition to the genes that we have identified, the bacterial symbiont *Wolbachia* also makes *D. melanogaster* more resistant to viral infection [43], [44]. *Wolbachia* occurs in natural populations, and protects flies against two of the viruses we studied — DCV and FHV [43], [44] (but not sigma viruses, Teixeira, Magwire and Wilfert, Pers. Comm.). As *Wolbachia* is transmitted vertically from mother to offspring, in many ways it can be regarded as another major-effect resistance polymorphism. We cured *Wolbachia* in our experiments with DCV and FHV and therefore have no data on its effects, but in natural populations of *D. melanogaster* it varies in prevalence from below 1% to near fixation [45]. This may affect how selection acts on the polymorphisms that we have identified, as our resistance genes may confer less of a benefit in populations where *Wolbachia* is common.

Will the genetic architecture of resistance to other classes of pathogens be similar to the pattern we have seen for viruses? In *Drosophila*, parasitoid wasps are one of the main causes of mortality in natural populations [46], and both linkage mapping and artificial selection experiments suggest that resistance against these parasites is controlled by a few major-effect loci [18], [47], [48]. There is also extensive variation in bacterial resistance, and polymorphisms in immune system genes explain a substantial proportion of this variation [49], [50]. It is difficult to compare these results directly to our own as comparatively few markers were genotyped and only known immune genes were investigated. Nonetheless, it would appear possible that bacterial resistance may have a more complex genetic architecture than virus resistance, involving more genes and epistatic interactions. Furthermore, there was not the clear difference between bacteria isolated from *D. melanogaster* and other organisms that we observed among our viruses [49], [50]. This may be because there is a broad-spectrum induced immune response against bacteria [51] but not viruses [52], or because the viruses may have a narrower host range, resulting in more rapid coevolution.

In the long term, the spread of the resistance genes through populations could either result in the virus evolving to overcome host resistance, or a permanent increase in the levels of resistance seen in the host population. Due to their high mutation rates, short generation times and large population sizes, RNA viruses can evolve rapidly [53]. Therefore, it is perhaps unsurprising that during the 1980s and 1990s sigma virus genotypes that were not affected by *ref(2)P* resistance spread through European populations of *D. melanogaster* [54]. This suggests that we may be observing one side of a coevolutionary arms race between hosts and parasites.

While the antimicrobial immune response of *Drosophila* is well-understood, we have only begun to understand in detail how *Drosophila* defends itself against viruses in the last six years [55]. Resistance to viruses could potentially evolve by altering the immune system (antiviral genes), or host factors that are usually exploited by viruses during the viral replication cycle (proviral genes). The only highly significant gene that we identified with a well-characterised function encodes ref(2)P, which is a homolog of the mammalian protein p62 [56]. This is a scaffold protein that has several functions, including in targeting cargoes such as protein aggregates and pathogens for destruction by autophagy — a process by which the cargo is wrapped in a double membrane vesicle called an autophagosome, which then fuses with the lysosome and is degraded [57], [58]. Autophagy was recently found to be an important component of antiviral immunity in *D. melanogaster* infected with vesicular stomatitis virus (VSV), which is another rhabdovirus that is related to DMelSV [24], [59]. Therefore, it is possible that this polymorphism is affecting the antiviral immune response. We also found suggestive evidence that a polymorphism in *scavenger receptor C1* may be important in defence against DAffSV. This gene functions both as a pattern recognition receptor of bacteria [30] and allows the uptake of dsRNA into cells, resulting in an RNAi response [31]. The function of this gene therefore suggests it is a strong candidate as a component of antiviral immunity.

The functions of the two genes that have the largest effects on susceptibility, *CHKov1* and *pastrel*, in antiviral defence remain unclear, although *pastrel* is thought to play a role in protein secretion [60]. Interestingly, knocking down the susceptible allele of this gene further increased susceptibility, suggesting that even the susceptible allele of the gene has some antiviral effect. Therefore, this gene may be part of the flies antiviral immune system. Characterising the role of *CHKov1* and *pastrel* in the immune system or the viral life cycle promises to yield new insights into both how animals evolve resistance to infection, and how viruses interact with their hosts.

## Methods

### Resistance assays

Many of the DGRP lines are infected with *Wolbachia* bacteria [28], which affects susceptibility to DCV and FHV [44]. To clear the stocks of *Wolbachia* infection, flies were reared for two generations on food prepared by adding 6 ml of 0.05% w/v tetracycline to a vial containing 5 g instant *Drosophila* medium (Carolina Biological, Burlington, North Carolina, U.S.A.) and yeast. We checked the flies were uninfected by PCR as described in reference [61]. Lab fly stocks can also be naturally infected by DCV, so the lines were also cleared of natural virus infections by aging adult flies for 20 days and then dechorionating embryos with a ∼5% sodium hypochlorite solution [16]. A small number of the lines assayed for DAffSV were not treated in this way, but excluding these lines did not alter the results. Note that the DMelSV data was collected previously, before the lines were treated [7] (*Wolbachia* does not affect sigma viruses, Teixeira, Magwire and Wilfert, Pers. Comm.).

The generation prior to injecting the viruses, vials were set up containing two male and two female flies on cornmeal-agar food. For each fly line/virus combination, we set up 4 vials whenever possible. To maximise the level of cross-factoring, we always used different combinations of fly lines on each day of injecting. The flies were injected with 69 nl of the virus suspension intra-abdominally as described in detail in references [62], [63]. The infection of *D. melanogaster* by DAffSV has not been characterised before, so we first injected flies with the virus and monitored viral titres and the characteristic symptom of sigma virus infection — sensitivity to CO_2_. These pilot experiments confirmed previous results that the virus can replicate in *D. melanogaster* [62], and also showed that infected flies are paralysed following exposure to CO_2_ (Figure S3).

Therefore, to assay for infection by DAffSV, flies were exposed to pure carbon dioxide for 15 min at 12°C at 15 days post-injection. By 30 min post-exposure, flies are awake from this anaesthesia were classed as uninfected, but flies that were dead or paralysed were classed as infected [24]. To assay for susceptibility to DCV and FHV we recorded survival every 24 hours until all the flies had died. Due to a historical accident, the DCV and FHV experiments used male flies and the DAffSV and DMelSV experiments used females. Therefore, care should be taken in comparing these datasets (note that these pairs of experiments also differ in the trait being measured). The DMelSV data has been published previously [7], [8]. We have genotyped the DGRP lines for the polymorphisms in *CHKov1* and *ref(2)P* by PCR as described in reference [7], [8].

We knocked down the *pastrel* gene by RNAi. Males from line UAS-*pst* (P{KK105159}VIE-260B) were crossed to *Actin*-GAL4 (w*;; P{GAL4-da.G32}UH1) virgins. As controls, males from a line with the same genetic background as UAS-*pst* (*y,w^1118^*;P{attP,*y^+^,w^3′^*}) and UAS-CG10669 (P{KK105150}VIE-260B) were also crossed to *Actin*-GAL4 virgins. F1 females were injected with DCV. We used two doses of DCV (high: TCID_50_ = 1000 and low: TCID_50_ = 690). We note that these were from different viral preparations, which may explain why this small difference in TCID_50_ caused a large difference in mortality.

We measured DCV titres by quantitative PCR using the SensiFAST™ SYBR & Fluorescein Kit (Bioline, UK). DCV was amplified using the primers DCV 6060F (5′-CTTGCGGACCCTTTGTACGAC-3′) and DCV 7320R (5′-GCCATTCGAACTTGACCACGCAG-3′). As an endogenous control we amplified *Actin5c* using the primers qActin5c_for2 (5′GAGCGCGGTTACTCTTTCAC 3′) and qActin5c_rev2 (5′ AAGCCTCCATTCCCAAGAAC 3′).We performed three technical replicates of each PCR and used the mean of these in subsequent analyses. We calculated the titre of DCV relative to *Actin5c* as 2^ΔCt^, where ΔCt is the critical threshold cycle of DCV minus the critical threshold cycle of *Actin5c*. This approach assumes near 100% primer efficiency, which was confirmed using a dilution series of the template cDNA.

### Analysis of genetic variation

We fitted a series of linear models to estimate genetic variances and covariances. For FHV and DCV, our data consisted of the lifespan of individual flies, which we treat as a Gaussian response in a general linear model. We fitted separate models for each virus, which were formulated as follows. Let *y_ijk_* be survival time (days after injection) of fly *k* from line *i* and vial *j*.
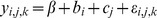
(1)where *β* is the mean survival time across all lines, *b_i_* is a random variable representing the deviation from the overall mean of the *i*th line, *c_j_* is a random variable representing the deviation *j*th vial from the line mean, and *ε_i,j,k_* is the residual error.

For DMelSV and DAffSV our data consist of numbers of infected and uninfected flies in each vial, which we treat as a binomial response in a generalized linear model. Let *v_i,k_* be the probability of flies in vial *k* from line *i* being infected.
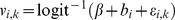
(2)where *β* is the overall mean, *b_i_* is a random variable representing the deviation from the overall mean of the *i*th line, and *ε_ik_* is a residual which captures over-dispersion within each vial due to unaccounted for heterogeneity between vials in the probability of infection.

To estimate the genetic correlations between the viruses, we analysed data from all four viruses using a single model. To allow us to treat data from all four viruses as a binomial response in a generalized linear model, for FHV and DCV we used the numbers of dead and alive flies on a single day. Let *v_i,j,k_* be the probability of flies, in vial *k* from line *i* and infected with virus *j* being dead.
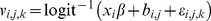
(3)where *β* is a vector of the mean survival times of the four virus types, and *x_i_* is a row vector relating this fixed effect to vial *i*. *b_i,j_* is the random effect of virus *k* on line *j*, and was assumed to be multivariate normally distributed, allowing us to estimate separate line variances for each virus type and covariances between all pairwise combinations of viruses. *ε_i,j,k_* is the residual which captures over-dispersion within each vial. The residuals were assumed to be normally distributed with a separate variance estimated for each virus type.

The parameters of the models were estimated using the R library MCMCglmm [64], which uses Bayesian Markov chain Monte Carlo (MCMC) techniques. Each model was run for 1.3 million iterations with a burn-in of 300,000, a thinning interval of 100 and improper priors. We confirmed these results were not influenced by the choice of prior in the Bayesian analysis by also fitting models 1 and 2 using maximum likelihood (data not shown). Credible intervals on variances, correlations, and heritability were calculated from highest posterior density intervals.

As these fly lines are homozygous across most genes in the genome, the genetic variance, *V_g_*, is half the between-line variance (assuming additive genetic variation). This allows us to calculate the heritability of DCV and FHV as:
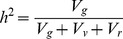
(4)Where *V_v_* is the between vial variance and *V_r_* is the residual variance. As the DMelSV and DAffSV parameters are on a logit scale, we calculated heritability as:
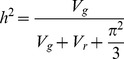
(5)where 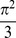 is the variance of a logistic distribution (the cumulative distribution function of the logistic distribution is the inverse logit function, the link function used in the model; [65], [66]). Note that the between-vial variance is included in *V_r_* in this model. In Table 1, we calculate *V_e_* as *V_r_*+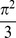. When calculating the proportion of the heritability explained by the polymorphisms we identified, we recalculated *V_g_* after accounting for these polymorphisms, and then adjusted the numerators of equations (4) and (5) accordingly.

We also calculated the coefficient of genetic variation, *CV_g_*, for DCV and FHV as 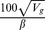, where β is the mean survival time [27]. To estimate the proportion of the heritability that is explained by these genes we assumed the polymorphisms have additive effects, so their contribution to additive genetic variation is 2*pqa* ^2^, where *p* and *q* are the frequencies of the alleles in the population used to calculate *h^2^*, and *a* is half the difference in the survival or infection probability of flies that are homozygous for the resistant and susceptible alleles (i.e. *a* and −*a* are the genotypic values of the resistant and susceptible homozygotes). The maximum likelihood estimate of *a*, 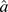, was obtained by regressing genotype against the line means during the GWAS (see below). An unbiased estimate of *a* ^2^ was obtained as 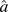 ^2^ minus the square of the standard error of 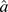. In this calculation, the heritability of resistance to DMelSV was recalculated from the line means which were treated as Gaussian data.

We used robust statistics to analyse data on viral titres due to the presence of an outlier in the data. We fitted a linear model by robust regression using an *M* estimator and used a robust *F* test to assess significance [67].

### Genome-wide association study

To identify single nucleotide polymorphisms (SNPs) that were associated to susceptibility, we performed a GWAS using the published DGRP genome sequences [28]. We only included biallelic SNPs where the minor allele occurred in at least 4 lines, and treated segregating sites within lines as missing data. In the case of DCV and FHV, the susceptibility of the line was measured as the mean survival time of flies in each line (with the vials of flies weighted by the number of flies in each vial). In the case of DMelSV and DAffSV, the susceptibility of the line was measured as the proportion of flies that were infected, as determined by the CO_2_ assay. The DAffSV data was arcsine square root transformed to remove the dependence of the variance on the mean. To each SNP we fitted the linear model *r_i,j_ = *β*+m_i_+ε_i_*, where *r_i,j_* is the susceptibility of flies with SNP genotype *i* from line *j*, β is the overall mean, *m_i_* is the SNP *i* genotype, and *ε_ik_* the residual. As major-effect polymorphisms affect the susceptibility of flies to DCV and DMelSV, the analysis was then repeated including the genotype of these genes as an additional explanatory variable.

Because we are performing multiple correlated tests, we determined a genome-wide significance threshold for the association between a SNP and phenotype by permutation. The phenotype data were permuted over the different recombinant lines, the genome-wide association study was repeated as described above, and the minimum *P-*value across the entire genome was recorded. This was carried out 400 times to generate a null distribution.

## Supporting Information

Figure S1 Manhattan plots of the *P*-values for the association between SNPs and virus resistance in the region of three resistance genes. The data is the same as Figure 2 in the main text. The arrows show the location of the genes (*pastrel*, *CHKov1*, *CHKov2* and *ref(2)P*). The blue box shows the location of the *doc* element insertion that is believed to increase resistance to DMelSV.
(PDF)

Figure S2 Results of the GWAS that included *ref(2)P* and *CHKov1* genotypes as fixed effects in the DMelSV model, and included *pastrel* as a fixed effect in the DCV model. The horizontal lines are genome-wide significance thresholds of *P* = 0.05 (solid line) and *P* = 0.2 (dashed line) that were obtained by permutation.
(PDF)

Figure S3 DAffSV replicates in *D. melanogaster*. The red circles shows the titre of DAffSV relative to *actin 5c* mRNA following injection of the virus. As is typical for sigma viruses, there is an initial drop in viral titre, presumably due to virions that were injected but do not infect cells. This is followed by an increase in titre as the virus replicates. The red line shows the predicted values from a second order polynomial regression. There was a significant effect of the second order term (*t* = 3.86, d.f. = 32, *p* = 0.0005). The blue triangles show the proportion of flies that were paralysed after exposure to CO_2_, and the blue line shows the predicted values from a second order polynomial regression (first order term: *t* = 4.29, d.f. = 32, *p* = 0.0002; second order term: *t* = 2.73, d.f. = 32, *p* = 0.01). Each data point is a vial of flies, with four vials per day and a mean of 16 flies/vial.
(PDF)

## References

[1] Tinsley MC, Blanford S, Jiggins FM (2006) Genetic variation in Drosophila melanogaster pathogen susceptibility. Parasitology 132: 767–773. 16497252 10.1017/S0031182006009929 PMC1847563

[2] Lazzaro B, Sceurman B, Clark A (2004) Genetic basis of natural variation in D-melanogaster antibacterial immunity. Science 303: 1873–1876. 15031506 10.1126/science.1092447

[3] Cooke GS, Hill AVS (2001) Genetics of susceptibility to human infectious disease. Nature Reviews Genetics 2: 967–977. 10.1038/35103577 11733749

[4] Thompson J, Burdon J (1992) Gene-for-gene coevolution between plants and parasites. Nature 360: 121–125.

[5] Woolhouse MEJ, Haydon DT, Antia R (2005) Emerging pathogens: the epidemiology and evolution of species jumps. Trends in Ecology & Evolution 20: 238–244. 16701375 10.1016/j.tree.2005.02.009 PMC7119200

[6] Woolhouse MEJ, Webster JP, Domingo E, Charlesworth B, Levin BR (2002) Biological and biomedical implications of the co-evolution of pathogens and their hosts. Nature Genetics 32: 569–577. 12457190 10.1038/ng1202-569

[7] Magwire MM, Bayer F, Webster CL, Cao C, Jiggins FM (2011) Successive Increases in the Resistance of Drosophila to Viral Infection through a Transposon Insertion Followed by a Duplication. PLoS Genet 7: e1002337 doi:10.1371/journal.pgen.1002337. 22028673 10.1371/journal.pgen.1002337 PMC3197678

[8] Bangham J, Obbard DJ, Kim KW, Haddrill PR, Jiggins FM (2007) The age and evolution of an antiviral resistance mutation in Drosophila melanogaster. Proceedings of the Royal Society B-Biological Sciences 274: 2027–2034. 10.1098/rspb.2007.0611 PMC1914336 17550883

[9] Stahl EA, Dwyer G, Mauricio R, Kreitman M, Bergelson J (1999) Dynamics of disease resistance polymorphism at the Rpm1 locus of Arabidopsis. Nature 400: 667–671. 10458161 10.1038/23260

[10] Visscher PM, Brown MA, McCarthy MI, Yang J (2012) Five Years of GWAS Discovery. American Journal of Human Genetics 90: 7–24. 22243964 10.1016/j.ajhg.2011.11.029 PMC3257326

[11] Pritchard JK (2001) Are rare variants responsible for susceptibility to complex diseases? American Journal of Human Genetics 69: 124–137. 11404818 10.1086/321272 PMC1226027

[12] Yang JA, Benyamin B, McEvoy BP, Gordon S, Henders AK, et al (2010) Common SNPs explain a large proportion of the heritability for human height. Nature Genetics 42: 565–U131. 20562875 10.1038/ng.608 PMC3232052

[13] Hill A (2012) Evolution, revolution and heresy in the genetics of infectious disease susceptibility. Philosophical Transactions of the Royal Society B-Biological Sciences 367: 840–849. 10.1098/rstb.2011.0275 PMC3267114 22312051

[14] Contamine D, Petitjean AM, Ashburner M (1989) Genetic resistance to viral infection - the molecular-cloning of a *Drosophila* gene that restricts infection by the rhabdovirus sigma. Genetics 123: 525–533. 2557263 10.1093/genetics/123.3.525 PMC1203824

[15] Luijckx P, Fienberg H, Duneau D, Ebert D (2012) Resistance to a bacterial parasite in the crustacean Daphnia magna shows Mendelian segregation with dominance. Heredity 108: 547–551. 22167056 10.1038/hdy.2011.122 PMC3330695

[16] Brun G, Plus N (1998) The viruses of *Drosophila* In: Ashburner M, Wright T, editors. The Genetics and Biology of *Drosophila*. London: Academic Press. pp. 625–700.

[17] Bangham J, Knott SA, Kim KW, Young RS, Jiggins FM (2008) Genetic variation affecting host-parasite interactions: major-effect quantitative trait loci affect the transmission of sigma virus in Drosophila melanogaster. Molecular Ecology 17: 3800–3807. 18665899 10.1111/j.1365-294X.2008.03873.x

[18] Poirie M, Frey F, Hita M, Huguet E, Lemeunier F, et al (2000) Drosophila resistance genes to parasitoids: chromosomal location and linkage analysis. Proceedings of the Royal Society of London Series B-Biological Sciences 267: 1417–1421. 10.1098/rspb.2000.1158 PMC1690685 10983825

[19] Wilfert L, Jiggins FM (2010) Disease association mapping in Drosophila can be replicated in the wild. Biology Letters 6: 666–668. 20444760 10.1098/rsbl.2010.0329 PMC2936165

[20] Lazzaro BP, Sackton TB, Clark AG (2006) Genetic variation in Drosophila melanogaster resistance to infection: A comparison across bacteria. Genetics 174: 1539–1554. 16888344 10.1534/genetics.105.054593 PMC1667071

[21] Sackton TB, Lazzaro BP, Clark AG (2010) Genotype and Gene Expression Associations with Immune Function in Drosophila. PLoS Genet 6: e1000797 doi:10.1371/journal.pgen.1000797. 20066029 10.1371/journal.pgen.1000797 PMC2793509

[22] Kapun M, Nolte V, Flatt T, Schlotterer C (2010) Host Range and Specificity of the Drosophila C Virus. PLoS ONE 5: e12421 doi:10.1371/journal.pone.0012421. 20865043 10.1371/journal.pone.0012421 PMC2928731

[23] Christian PD (1987) Studies on Drosophila C and A viruses in Australian populations of *Drosophila melanogaster*: Australian National University.

[24] Longdon B, Obbard DJ, Jiggins FM (2010) Sigma viruses from three species of Drosophila form a major new clade in the rhabdovirus phylogeny. Proceedings of the Royal Society B-Biological Sciences 277: 35–44. 10.1098/rspb.2009.1472 PMC2842628 19812076

[25] Longdon B, Wilfert L, Osei-Poku J, Cagney H, Obbard DJ, et al (2011) Host-switching by a vertically transmitted rhabdovirus in Drosophila. Biology Letters 7: 747–750. 21450721 10.1098/rsbl.2011.0160 PMC3169049

[26] Price B, Rueckert R, Ahlquist P (1996) Complete replication of an animal virus and maintenance of expression vectors derived from it in Saccharomyces cerevisiae. Proceedings of the National Academy of Sciences of the United States of America 93: 9465–9470. 8790353 10.1073/pnas.93.18.9465 PMC38451

[27] Houle D (1992) Comparing evolvability and variability of quantitative traits. Genetics 130: 195–204. 1732160 10.1093/genetics/130.1.195 PMC1204793

[28] Mackay TFC, Richards S, Stone EA, Barbadilla A, Ayroles JF, et al (2012) The Drosophila melanogaster Genetic Reference Panel. Nature 482: 173–178. 22318601 10.1038/nature10811 PMC3683990

[29] Nakamura N (1978) Dosage effects of non-permissive allele of *Drosophila* ref(2)P gene on sensitive strains of sigma virus. Molecular & General Genetics 159: 285–292. 416337 10.1007/BF00268264

[30] Ramet M, Pearson A, Manfruelli P, Li XH, Koziel H, et al (2001) Drosophila scavenger receptor Cl is a pattern recognition receptor for bacteria. Immunity 15: 1027–1038. 11754822 10.1016/s1074-7613(01)00249-7

[31] Ulvila J, Parikka M, Kleino A, Sormunen R, Ezekowitz RA, et al (2006) Double-stranded RNA is internalized by scavenger receptor-mediated endocytosis in Drosophila S2 cells. Journal of Biological Chemistry 281: 14370–14375. 16531407 10.1074/jbc.M513868200

[32] Peradziryi H, Kaplan N, Podleschny M, Liu X, Wehner P, et al (2011) PTK7/Otk interacts with Wnts and inhibits canonical Wnt signalling. Embo Journal 30: 3729–3740. 21772251 10.1038/emboj.2011.236 PMC3173783

[33] Beavis WD (1998) QTL analyses: power, precision, and accuracy. In: Paterson AH, editor. Molecular Dissection of Complex Traits. New York: CRC Press. pp. 145–162.

[34] Aminetzach YT, Macpherson JM, Petrov DA (2005) Pesticide resistance via transposition-mediated adaptive gene truncation in Drosophila. Science 309: 764–767. 16051794 10.1126/science.1112699

[35] Chapman S, Hill A (2012) Human genetic susceptibility to infectious disease. Nature Reviews Genetics 13: 175–188. 10.1038/nrg3114 22310894

[36] Wilfert L, Schmid-Hempel P (2008) The genetic architecture of susceptibility to parasites. Bmc Evolutionary Biology 8. 10.1186/1471-2148-8-187 PMC2446395 18590517

[37] Wayne ML, Contamine D, Kreitman M (1996) Molecular population genetics of ref(2)P, a locus which confers viral resistance in Drosophila. Molecular Biology and Evolution 13: 191–199. 8583891 10.1093/oxfordjournals.molbev.a025555

[38] Barton N, Turelli M (1987) Adaptive landscapes, genetic-distance and the evolution of quantitative characters. Genetical Research 49: 157–173. 3596236 10.1017/s0016672300026951

[39] Blows M, Hoffmann A (2005) A reassessment of genetic limits to evolutionary change. Ecology 86: 1371–1384.

[40] Reeve J (2000) Predicting long-term response to selection. Genetical Research 75: 83–94. 10740924 10.1017/s0016672399004140

[41] Blows M, Higgie M (2003) Genetic constraints on the evolution of mate recognition under natural selection. American Naturalist 161: 240–253. 10.1086/345783 12675370

[42] McKenzie J, Batterham P (1998) Predicting insecticide resistance: mutagenesis, selection and response. Philosophical Transactions of the Royal Society of London Series B-Biological Sciences 353: 1729–1734. 10.1098/rstb.1998.0325 PMC1692398 10021773

[43] Hedges L, Brownlie J, O'Neill S, Johnson K (2008) Wolbachia and Virus Protection in Insects. Science 322: 702–702. 18974344 10.1126/science.1162418

[44] Teixeira L, Ferreira A, Ashburner M (2008) The Bacterial Symbiont Wolbachia Induces Resistance to RNA Viral Infections in Drosophila melanogaster. PLoS Biol 6: e1000002 doi:10.1371/journal.pbio.1000002. 10.1371/journal.pbio.1000002 PMC2605931 19222304

[45] Verspoor R, Haddrill P (2011) Genetic Diversity, Population Structure and Wolbachia Infection Status in a Worldwide Sample of Drosophila melanogaster and D. simulans Populations. PLoS ONE 6: e26318 doi:10.1371/journal.pone.0026318. 22022599 10.1371/journal.pone.0026318 PMC3192181

[46] Fleury F, Ris N, Allemand R, Fouillet P, Carton Y, et al (2004) Ecological and genetic interactions in Drosophila-parasitoids communities: a case study with D-melanogaster, D-simulans and their common Leptopilina parasitoids in south-eastern France. Genetica 120: 181–194. 15088657 10.1023/b:gene.0000017640.78087.9e

[47] Kraaijeveld A, Godfray H (1997) Trade-off between parasitoid resistance and larval competitive ability in Drosophila melanogaster. Nature 389: 278–280. 9305840 10.1038/38483

[48] Hita M, Espagne E, Lemeunier F, Pascual L, Carton Y, et al (2006) Mapping candidate genes for Drosophila melanogaster resistance to the parasitoid wasp Leptopilina boulardi. Genetical Research 88: 81–91. 17125583 10.1017/S001667230600841X

[49] Lazzaro BP, Sceurman BK, Clark AG (2004) Genetic basis of natural variation in D-melanogaster antibacterial immunity. Science 303: 1873–1876. 15031506 10.1126/science.1092447

[50] Lazzaro B, Sackton T, Clark A (2006) Genetic variation in Drosophila melanogaster resistance to infection: A comparison across bacteria. Genetics 174: 1539–1554. 16888344 10.1534/genetics.105.054593 PMC1667071

[51] Lemaitre B, Hoffmann J (2007) The host defense of Drosophila melanogaster. Annual Review of Immunology 25: 697–743. 10.1146/annurev.immunol.25.022106.141615 17201680

[52] Carpenter J, Hutter S, Baines JF, Roller J, Saminadin-Peter SS, et al (2009) The Transcriptional Response of Drosophila melanogaster to Infection with the Sigma Virus (Rhabdoviridae). PLoS ONE 4: e6838 doi:10.1371/journal.pone.0006838. 19718442 10.1371/journal.pone.0006838 PMC2730013

[53] Holmes EC (2009) The Evolution and Emergence of RNA Viruses [electronic resource]. Oxford: OUP Oxford. 1 online resource (267 p.) p.

[54] Fleuriet A, Sperlich D (1992) Evolution of the *Drosophila-melanogaster*-sigma virus system in a natural population from Tubingen. Theoretical and Applied Genetics 85: 186–189. 24197303 10.1007/BF00222858

[55] Kemp C, Imler JL (2009) Antiviral immunity in drosophila. Current Opinion in Immunology 21: 3–9. 19223163 10.1016/j.coi.2009.01.007 PMC2709802

[56] Nezis IP, Simonsen A, Sagona AP, Finley K, Gaumer S, et al (2008) Ref(2) P, the Drosophila melanogaster homologue of mammalian p62, is required for the formation of protein aggregates in adult brain. Journal of Cell Biology 180: 1065–1071. 18347073 10.1083/jcb.200711108 PMC2290837

[57] Korolchuk VI, Mansilla A, Menzies FM, Rubinsztein DC (2009) Autophagy Inhibition Compromises Degradation of Ubiquitin-Proteasome Pathway Substrates. Molecular Cell 33: 517–527. 19250912 10.1016/j.molcel.2009.01.021 PMC2669153

[58] Lamark T, Kirkin V, Dikic I, Johansen T (2009) NBR1 and p62 as cargo receptors for selective autophagy of ubiquitinated targets. Cell Cycle 8: 1986–1990. 19502794 10.4161/cc.8.13.8892

[59] Shelly S, Lukinova N, Bambina S, Berman A, Cherry S (2009) Autophagy Is an Essential Component of Drosophila Immunity against Vesicular Stomatitis Virus. Immunity 30: 588–598. 19362021 10.1016/j.immuni.2009.02.009 PMC2754303

[60] Bard F, Casano L, Mallabiabarrena A, Wallace E, Saito K, et al (2006) Functional genomics reveals genes involved in protein secretion and Golgi organization. Nature 439: 604–607. 16452979 10.1038/nature04377

[61] Jiggins F, Hurst G, Majerus M (2000) Sex-ratio-distorting Wolbachia causes sex-role reversal in its butterfly host. Proceedings of the Royal Society B-Biological Sciences 267: 69–73. 10.1098/rspb.2000.0968 PMC1690502 10670955

[62] Longdon B, Hadfield JD, Webster CL, Obbard DJ, Jiggins FM (2011) Host phylogeny determines viral persistence and replication in novel hosts. PLoS Pathog 7: e1002260 doi:10.1371/journal.ppat.1002260. 21966271 10.1371/journal.ppat.1002260 PMC3178573

[63] Longdon B, Fabian D, Hurst G, Jiggins F (2012) Male-killing Wolbachia do not protect Drosophila bifasciata against viral infection. BMC Microbiology 12: S8. 22376177 10.1186/1471-2180-12-S1-S8 PMC3287519

[64] Hadfield JD (2010) MCMC Methods for Multi-Response Generalized Linear Mixed Models: The MCMCglmm R Package. Journal of Statistical Software 33: 1–22. 20808728 PMC2929880

[65] Lee Y, Nelder JA (2001) Hierarchical generalised linear models: A synthesis of generalised linear models, random-effect models and structured dispersions. Biometrika 88: 987–1006.

[66] Nakagawa S, Schielzeth H (2010) Repeatability for Gaussian and non-Gaussian data: a practical guide for biologists. Biological Reviews 85: 935–956. 20569253 10.1111/j.1469-185X.2010.00141.x

[67] Huber PJ (1981) Robust statistics. New York ; Chichester: Wiley. ix,308p. p.

