# Supplementary figures and images for: Genome-Wide Association Studies Reveal a Simple Genetic Basis of Resistance to Naturally Coevolving Viruses in Drosophila melanogaster
Source: PLoS Genet. 2012 Nov 15;8(11):e1003057. doi: 10.1371/journal.pgen.1003057 (PMC3499358; doi:10.1371/journal.pgen.1003057)

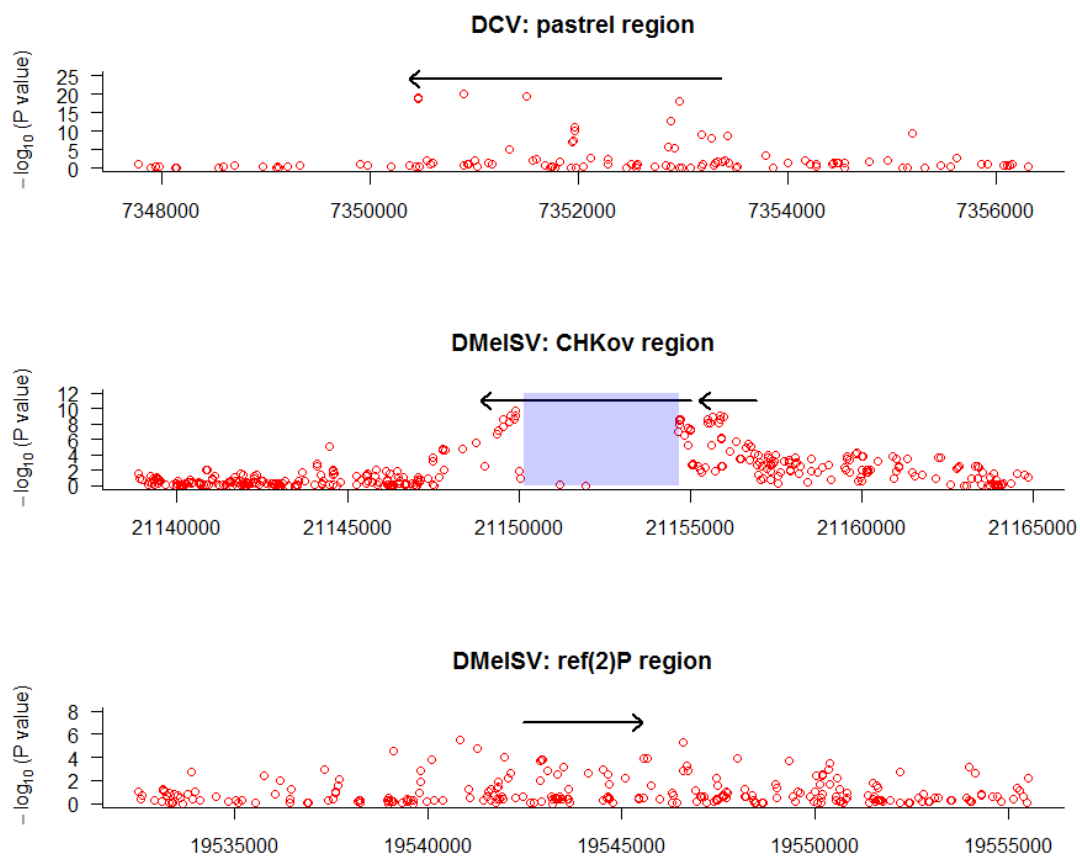

Supplement: Figure S1 — Manhattan plots of the P-values for the association between SNPs and virus resistance in the region of three resistance genes. The data is the same as Figure 2 in the main text. The arrows show the location of the genes (pastrel, CHKov1, CHKov2 and ref(2)P). The blue box shows the location of the doc element insertion that is believed to increase resistance to DMelSV. (PDF) [file pgen.1003057.s001.pdf]

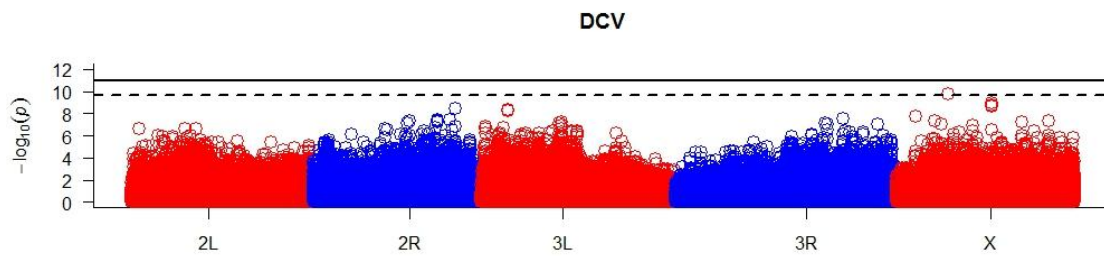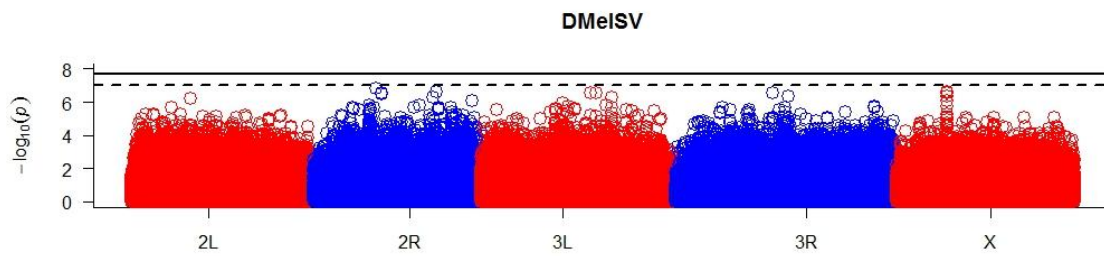

Supplement: Figure S2 — Results of the GWAS that included ref(2)P and CHKov1 genotypes as fixed effects in the DMelSV model, and included pastrel as a fixed effect in the DCV model. The horizontal lines are genome-wide significance thresholds of P = 0.05 (solid line) and P = 0.2 (dashed line) that were obtained by permutation. (PDF) [file pgen.1003057.s002.pdf]

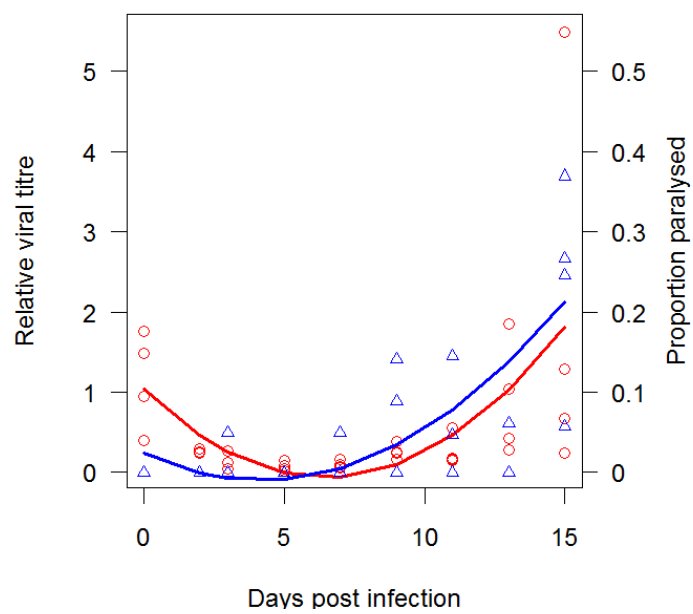

Supplement: Figure S3 — DAffSV replicates in D. melanogaster. The red circles shows the titre of DAffSV relative to actin 5c mRNA following injection of the virus. As is typical for sigma viruses, there is an initial drop in viral titre, presumably due to virions that were injected but do not infect cells. This is followed by an increase in titre as the virus replicates. The red line shows the predicted values from a second order polynomial regression. There was a significant effect of the second order term (t = 3.86, d.f. = 32, p = 0.0005). The blue triangles show the proportion of flies that were paralysed after exposure to CO2, and the blue line shows the predicted values from a second order polynomial regression (first order term: t = 4.29, d.f. = 32, p = 0.0002; second order term: t = 2.73, d.f. = 32, p = 0.01). Each data point is a vial of flies, with four vials per day and a mean of 16 flies/vial. (PDF) [file pgen.1003057.s003.pdf]
